# Breastfeeding and infant growth outcomes in the context of intensive peer counselling support in two communities in Bangladesh

**DOI:** 10.1186/s13006-016-0077-6

**Published:** 2016-07-07

**Authors:** Rukhsana Haider, Kuntal Kumar Saha

**Affiliations:** Training & Assistance for Health & Nutrition Foundation (TAHN), Banani, Dhaka Bangladesh; Department of Nutrition for Health and Development, World Health Organization, Geneva, Switzerland

**Keywords:** Community-based peer counsellors, Exclusive breastfeeding, Low birth weight, Normal birth weight, Growth, Bangladesh

## Abstract

**Background:**

Exclusive breastfeeding and growth faltering during infancy remain challenges in Bangladesh. The Training & Assistance for Health & Nutrition Foundation has been working to address this gap through community-based peer counsellors since 2000. In this paper, we assessed the programme’s progress, particularly with respect to early initiation of breastfeeding and exclusive breastfeeding for normal birth weight, as well as for low birth weight (LBW) infants.

**Methods:**

The peer counselling programme is continuing in rural and urban areas of Bangladesh, but only data collected between January 2013 and June 2015 is presented in this descriptive study. Intensive breastfeeding counselling was provided to women during the third trimester of pregnancy and 6 months postpartum by well-trained peer counsellors. They recorded data on infants’ birth weight, feeding practices and body weight every month and submitted these for computer entry. Weight measurements were converted to weight-for-age Z-scores (WAZ) and growth patterns assessed by comparing attained body weights with the World Health Organization (WHO) 2006 Child Growth Standards.

**Results:**

A total of 994 infants were born during the study period; 94 % were normal birth weight and 6 % were LBW (<2.5 kg). Initiation of breastfeeding within one hour of birth was reported by 94 % of counselled mothers in both groups. The prevalence of exclusive breastfeeding at 6 months was 94 % in the normal birth weight infants and 92 % in the LBW infants, and their mean body weights were 7.5 ± 0.8 kg and 6.6 ± 0.7 kg respectively. There was no growth faltering in these infants during 6 months. Underweight rates in normal birth weight infants remained similar (2.2 % at 1 month and 2.5 % at 6 months), whereas underweight rates in LBW infants decreased from 42.1 % at 1 month to 21.1 % at 6 months.

**Conclusions:**

In the context of a well-structured programme setting, and under the described circumstances, it seems likely that the well-trained and supervised community-based peer counsellors could assist in encouraging and helping mothers of both normal birth weight and LBW infants to initiate breastfeeding within one hour and to continue exclusive breastfeeding until 6 months of age. Our data suggest that they may also have contributed towards prevention of growth faltering in these infants.

## Background

Optimal breastfeeding under 2 years of age has the greatest impact on child survival with the potential to prevent 13 % of all deaths in children under 5 years [1]. Systematic reviews have shown that exclusive breastfeeding for 6 months compared to exclusive breastfeeding for 3 to 4 months, can decrease diarrheal morbidity and prolong lactational amenorrhea while incurring no growth deficit among infants either in low or high income countries [2, 3]. In addition to the short-term effects of breastfeeding on reduction of morbidity and mortality [4, 5] and improvement in cognitive and brain development [6, 7], recent evidence further highlights the long-term effects on intelligence, educational attainment and income [8–10].

Breastfeeding promotion was initiated in Bangladesh in 1989, focusing initially on meetings with health professionals. It was followed by implementation of the Baby-Friendly Hospital Initiative in maternity hospitals, and intensified after the World Health Organization’s recommendation in 2002 to breastfeed infants exclusively for the first 6 months of life [11]. For several years however, the national prevalence of exclusive breastfeeding was stagnant, around 40 %, then rose suddenly to 64 % in 2011 [12] but fell soon after to 55 % in 2014 [13]. Although the under-five child malnutrition rates are decreasing slowly, they are still high with 36 % stunted, 14 % wasted and 33 % underweight children in 2014 [13].

LBW rates on the other hand, continue to be high. While the only national survey that recorded birth weights in 2003–4 reported that 36 % of infants were born LBW and 77 % had intrauterine growth retardation [14], a report from the urban slums of Dhaka showed the LBW prevalence was 46 % with 70 % small for gestational age (SGA) and 17 % preterm infants [15]. Recently, routine surveillance from a northern part of the country has documented even higher LBW rates of 55 % with 22 % preterm births [16].

Community-based breastfeeding peer counselling has been successful in increasing exclusive breastfeeding rates in Bangladesh [17] and elsewhere [18, 19]. Training & Assistance for Health & Nutrition Foundation (TAHN) was established in 2000 to enable the peer counsellors to continue working after our initial research [17] was completed, and to provide training on breastfeeding promotion and support to government and non-government organisations. Since the last few years however, the peer counsellors have been mentioning that although the LBW infants were given more attention, they still did not grow as well as the normal birth weight ones. A literature search showed a study from the Philippines [20] had selected term LBW infants in hospital and provided follow-up counselling and support to improve breastfeeding practices, but we found no reports that supported mothers of LBW babies at the community/household level. While acknowledging that effective breastfeeding promotion can improve breastfeeding patterns, the effects on growth are not always clear or conclusive [21–23]. Recognising that LBW infants can have unique breastfeeding problems and may have increased health risks [24], including delayed initiation that could increase neonatal mortality [25] and considering our own peer counsellors’ observations about problems with LBW babies, we decided to assess if, after counselling, there were any differences regarding their breastfeeding outcomes. The primary objective of this descriptive study thus was to examine if peer counsellors were continuing to have an impact on early initiation and exclusive breastfeeding practices of infants in their coverage areas (both normal birth weight and LBW), and if these practices could also improve the infants’ growth. The ongoing programme setting provided an excellent opportunity to do so.

## Methods

### Setting

The setting for this study was TAHN Foundation’s programme areas in *Badda*, Dhaka city, and *Anowara*, a rural sub-district of Chittagong.

### Study design

This was a descriptive study, with the aim of documenting process, outputs and outcomes related to community-based peer counselling and breastfeeding.

### Selection and training of peer counsellors

The peer counsellors are married women who have breastfed their own babies for at least 1 year (not necessary to have exclusively breastfed), have minimum 8 years of formal schooling, have lived in the same community for several years and are motivated to help other women improve their infant and child feeding and caring practices. They were trained using adapted, simplified versions of the WHO/UNICEF breastfeeding counselling course [26] as has been described elsewhere [27] and the national infant and young child feeding (IYCF) training guidelines [28]. In brief, the “classroom” breastfeeding training was conducted for four hours per day over 4 days, using locally adapted training materials. Practical demonstrations were held for counselling and communication skills with emphasis on; position and attachment for breastfeeding, encouraging exclusive breastfeeding, expression of breastmilk and feeding babies breastmilk with a small cup, and for breastfeeding related problem solving. The trainees were then trained “hands-on” for 6 days in their communities, counselling pregnant women and mothers of infants 0–6 months of age on early initiation and exclusive breastfeeding. After the peer counsellors had enrolled some mothers in the programme, the supervisors monitored at least 10 % of the peer counsellors’ counselling sessions using a pre-tested checklist and also took 10 % of mothers’ interviews for their feedback in the absence of the peer counsellors. During monthly meetings with peer counsellors, problems in the field were discussed. Refresher training was provided on quarterly basis for specific gaps in peer counselling, (e.g. position and attachment of LBW babies at mother’s breast, demonstration of manual expression of breastmilk, identification and management of common problems etc.)

### Programme implementation

Peer counsellors worked part time for the programme (3–4 h a day), and were paid an honorarium of Bangladeshi Taka 3,500–4,700 (approx. equivalent to USD 45–60) per month, depending upon their experience in the programme. Each peer counsellor could provide services to about 50–60 pregnant and lactating mothers per month (with infants in different age groups who were followed up until 18 months). The catchment area for each peer counsellor was designated as households within 30 min walking distance from her residence. During 2012–2015, there were four peer counsellors in *Badda* and five in *Anowara*.

The peer counsellors enrolled pregnant women in the last trimester of pregnancy after receiving informed verbal consent and visited them at least two times during that period. Table 1 lists specific tasks that peer counsellors performed during home visits to pregnant women. During early infancy, peer counsellors visited mothers three times during the first month (within 2 days after delivery, between 7 and 10 days, and at 1 month) and thereafter on average, once a month until the infants were 6 months old. If the mother or infant required more support, additional visits were also undertaken. The specific tasks that peer counsellors performed during postpartum home visits are shown in Table 2.

**Table 1:** Specific tasks of peer counsellors and key counselling topics during pregnancy

• Enrol pregnant women in the programme in the third trimester of pregnancy • Encourage them to go to health facility for regular antenatal checks and tetanus toxoid injections and; o Increase food intake at each meal and eat nutritious snacks in between` o Rest for about 2 h after lunch o Avoid lifting heavy weights • Encourage delivery at health facility or by trained/skilled birth attendant at home • Explain o Importance of early initiation of breastfeeding o Problems of pre-lacteal feeding o Benefits of exclusive breastfeeding o Dangers of feeding a baby non-human milk and of bottle feeding • Demonstrate correct position and attachment at the breast (with towel/doll) • Encourage washing hands before breastfeeding

**Table 2:** Specific tasks of peer counsellors and key counselling messages during first 6 months

• Remind mothers about o Benefits of exclusive breastfeeding o Dangers of non-human milk and of bottle feeding o Washing hands before breastfeeding • Observe position and attachment at the breast and correct if required • Check if mother understands how she will know that baby is getting sufficient breastmilk • Emphasise correct usage of lactation amenorrhoea method (LAM) for contraception and other methods if and when indicated • Demonstrate and check expression of breastmilk and feeding baby with small cup • Praise and encourage to continue exclusive breastfeeding	

Influential family members, particularly grandmothers and fathers, were the secondary target group for the peer counsellors to promote optimal breastfeeding practices. Family members were requested to share the household chores so that mothers could give more time for caring and feeding the infant. The peer counsellors also motivated mothers to adopt appropriate hygiene practices and health seeking behaviour, such as for antenatal care, timely immunization of mother and child, and early treatment of any illness. Through the TAHN office, they provided mothers with Vitamin A supplements (200,000 IU units) soon after delivery, advised them to buy iron and folic acid tablets (they were provided only if she could not afford to buy) and to continue taking these until infants were 3 months old. Kangaroo care [24] was demonstrated to mothers with infants below 2000 g and they were encouraged to continue using this method until babies weighed 2500 g. If there was a specific problem that the peer counsellor could not handle, she referred the mother either to a health facility (with a referral slip) or to the programme supervisors.

### Sample size and sampling

Using an exclusive breastfeeding rate of 32 % for 4–5 month old infants, estimated from the national Bangladesh Demographic and Health Survey of 2014 [13], and expecting an increase of at least 48 % in the peer counselled mothers (to reach 80 % exclusive breastfeeding) with 80 % power, we calculated a sample size of about 200 mothers. As we planned to study all the mothers who had been available in the programme during the period specified, and as we had more mothers than were needed for the analysis, we thought further calculations for a design effect were not necessary. But if we calculate for a design effect of 2, the total number would come to 400 mothers who had completed 6 months of follow-up.

Since we wanted to investigate what impact peer counsellors were having on breastfeeding practices, all households with pregnant women in a peer counsellor’s catchment area were included in the programme. A peer counsellor’s area covered approximately 4,000 households in the urban slums in *Badda*, Dhaka city and 2,450 households in rural *Anowara*. From January 2013 to June 2015, a total of 1838 mothers were counselled, but only babies born during that period with recorded birth weight, and who had completed 6 months follow-up were included in this study. Mothers were excluded because of out-migration, if they had more than three living children, resided more than one hour away from the peer counsellor’s house, refused peer counsellor’s services, or if their infants had died. Also excluded were twins and infants with suspected congenital problems.

### Infant feeding practices

Information about infant feeding practices was collected by the peer counsellors every month and recorded in their registers. Feeding practices were based on 24-h recall during a particular visit. Continued exclusive breastfeeding from birth to 6 months was recorded if at each consecutive visit and between visits, the mothers reported not having given anything but breastmilk to their babies. If water, honey, sugar-water etc. were fed in the first 3 days of life or later on, infants were labelled as predominantly breastfed [29], or if other milk or solids were given, as partially breastfed [30].

### Weight measurements

Birth weights and monthly weights of infants were measured using electronic scales with 100 g precision (Tanita, Japan). Birth weights were recorded at home within 2 days of birth. A mother and her infant were first weighed together, and then the mother alone to obtain the infant’s weight by subtraction. For each measurement, at least two readings were taken and the average weight noted. Standardisation of the weighing scales was performed whenever the supervisors went for field visits.

### In-depth interviews

Staff conducted in-depth interviews (over phone or in person) with mothers of infants who had been partially breastfed at any time during 6 months with the intention of identifying the main reasons and difficulties that had may have led to discontinuation of exclusive breastfeeding.

### Data analysis

Data about timing of initiation of breastfeeding and breastfeeding status on 24-h recall at each monthly visit were analysed. Timing of initiation of breastfeeding was estimated as the length of time in minutes and hours between delivery and the actual time when breastfeeding had started. Prevalence of exclusive breastfeeding among infants was ascertained from the reports as described earlier.

Weight measurements were converted to weight-for-age Z-scores (WAZ) according to the WHO 2006 Child Growth Standards [31]. Underweight was defined as < -2 Z-score of WAZ. Growth patterns of boys and girls (normal birth weight and LBW) were examined by comparing their attained monthly weights with the WHO 2006 Child Growth Standards.

Data were analysed using SPSS Version 17 (SPSS Inc., Chicago, USA). Descriptive analyses included frequencies and contingency tables, means and standard deviations. Statistical tests were not used to compare LBW with normal birth weight infants and mothers since the numbers in the former group were comparatively very small.

## Results

### General characteristics of the sample

**Fig. 1:**
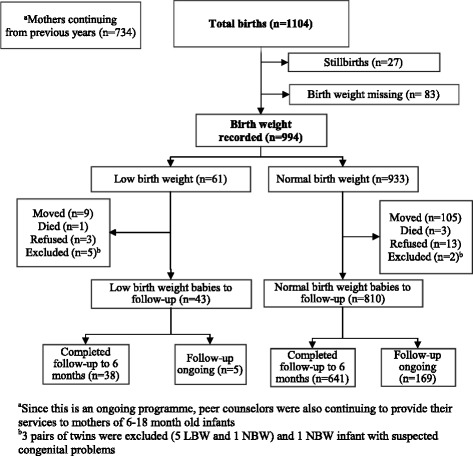
Flow chart of study participants in the programme area

A total of 1104 babies were born over the 2 and a half years between January 2013 and June 2015, among whom birth weights were recorded for 994 (Fig. 1). About 6 % of the babies were LBW (< 2.5 kg). Only 1.6 % of mothers had refused peer counsellor’s services, either because they/family preferred to get nutrition/ infant feeding advice from a doctor, or they did not want any outsider to visit their homes.

Mean birth weight of the normal birth weight infants was 3.1 ± 0.3 kg and that of the LBW infants was 2.1 ± 0.2 kg. There were no differences in the mean age, number of previous children, place of delivery, and health personnel who conducted the delivery among mothers of the normal birth weight and LBW infants (Table 3). Only the percentage of mothers of LBW infants who had no education was higher (13 %) compared to mothers of normal birth weight infants (5 %), but their numbers were small. About half of the mothers of normal birth weight infants delivered at health facilities (53 %), compared to the mothers of LBW infants (60 %). Similarly, a higher proportion of mothers of LBW infants delivered before 37 weeks of gestation, with more Caesarean sections (26 %) than mothers of normal birth weight infants (21 %).

**Table 3:** General characteristics of mothers and infants in the programme

	Normal birth weight	Low birth weight
	*n* = 641	*n* = 38
	Mean ± SD (Range)	Mean ± SD (Range)
Infant Birth weight (Kg)	3.1 ± 0.34 (2.5–4.4)	2.1 ± 0.23 (1.5–2.4)
Mothers age (years)	22.2 ± 3.3 (16–35)	23.1 ± 4.0 (18–33)
Mothers education		
Years of schooling	7.1 ± 3.1	6.5 ± 3.5
	%	%
No education	5.3	13.2
With education	94.7	86.8
Previous children		
0	59.1	50.0
≥ 1	40.9	50.0
Previous child was EBF		
Yes	95.8	68.4
No	4.2	31.6
Gestational age (weeks)^a^		
< 37	3.0	13.2
> 37	97.0	86.8
Place of delivery		
Health facility	53.5	60.5
Home	46.5	39.5
Delivery type		
Normal vaginal	78.5	73.7
Caesarean section	21.5	26.3
Delivered by		
Doctor/Nurse	52.3	63.2
TBA	47.7	36.8

*EBF* = exclusively breastfed, ^a^Expected date of delivery was missing for 3 normal birth weight infants, *TBA* = traditional birth attendant

### Breastfeeding practices

Reported breastfeeding initiation within one hour of birth was high for all the infants, about 94 % in both the normal birth weight and LBW infants (Table 4). Initiation was delayed when the infants were delivered by Caesarean section, and/or infants were separated from their mothers in the health facility, or were perceived to be too weak to suckle at the breast. Exclusive breastfeeding rates at 6 months using 24-h recall (94 % for normal birth weight and 92 % for LBW infants) were similar to those obtained by using post-delivery and consecutive monthly feeding practices data for 6 months (91 % for normal birth weight and 92 % for LBW infants).

**Table 4:** Initiation of breastfeeding and prevalence of exclusive breastfeeding in normal and low birth weight infants

	Normal birth weight	Low birth weight
	*n* = 641	*n* = 38
	(%)	(%)
Initiation of breastfeeding		
Within 1 h of birth	93.9	94.7
Within 3 days or more	6.1	5.3
Feeding Status at 6 months		
EBF^a^	93.8	92.1
Predominant breastfeeding	0.2	2.6
Partial breastfeeding	5.9	5.3
No breastfeeding	0.2	0.0
Feeding Status, birth to 6 months		
EBF^b^	91.3	92.1
Predominant breastfeeding	0.3	2.6
Partial breastfeeding	8.3	5.3
No breastfeeding	0.2	0

^a^ Using 24-h recall at infants’ completion of 6 months visit

^b^ Using 24-h recall within 3 days and at every monthly visit from 1 to 6 months

Fifty-three mothers of the normal birth weight infants had fed their infants other milk at some time during the 6 months, but none of the mothers of the LBW infants had fed anything else. The previous group of mothers were asked (through staff visits/phone calls) the reasons for this supplementation (data provided in Table 5). Apart from a small number of infants who were separated from their mothers in the first 3 days, and mothers being very unwell during the first month (27 %), the commonest answer was that the grandparents wanted to feed the baby other milk (17 %). In 30 % cases, doctors had advised to add other milk, whereas mothers themselves took the decision to do so in 43 % of cases, and other family members in 6 % of cases.

**Table 5:** Reasons and decision makers for adding other milk along with breastmilk in normal birth weight infants

	Reasons for adding other milk	Who decided/ started	No. and (%) of mothers
1	Added within 1^st^ 3 days after C/Sec^a^, as mother separated from baby	Doctor	3 (6)
2	Added within 1^st^ 3 days of normal delivery, as mother was very sick	Family members	3 (6)
3	C/Sec^a^ followed by pain in abdomen and body for many days. Mother unable to care for baby properly, who cried a lot	Doctor	8 (15)
4	Baby sick *(cough, fever, breathing problem, diarrhoea)*	Doctor	5 (9)
5	Baby cried at night	Pharmacy salesman	2 (4)
6	Wanted to feed baby other milk along with breastmilk	Grandparents	9 (17)
7	Mother thought she had insufficient milk	Mother	7 (13)
8	Mother and others thought baby did not want breastmilk	Mother	7 (13)
9	Mother was sick *(chicken pox, high fever)*	Mother	3 (6)
10	Mother went out for work *(factory, housemaid)*	Mother	4 (7)
11	Family problems *(quarrels & separation)*	Mother	2 (4)
TOTAL			53 (100)

^a^ C/Sec: Caesarean section

### Growth patterns and underweight prevalence in normal birth weight and LBW infants

**Fig. 2:**
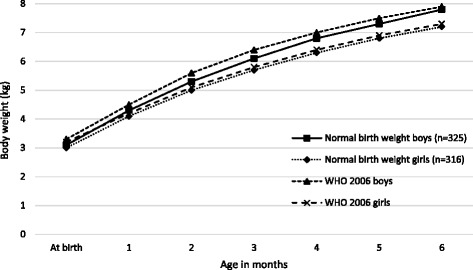
Comparison of body weight of normal birth weight infants with WHO child growth standards

**Fig. 3:**
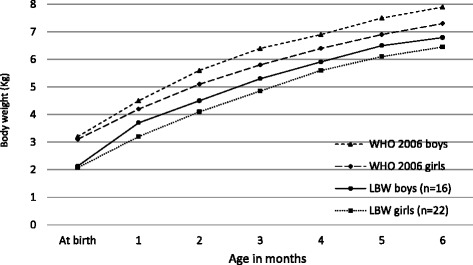
Comparison of body weight of low birth weight infants with WHO child growth standards

At 6 months, the mean body weight of normal birth weight boys was 7.8 ± 0.8 kg, compared to 6.8 ± 0.8 kg for the LBW boys. Likewise, at 6 months, the mean body weights were 7.2 ± 0.8 kg for normal birth weight girls and 6.5 ± 0.6 kg for the LBW girls. Initially the normal birth weight and LBW boys and girls grew parallel to, but in a lower track than the infants in the WHO growth standards (Figs. 2 and 3), but by 5 months the gap between the normal birth weight girls and those in the WHO standards had almost disappeared.

**Fig. 4:**
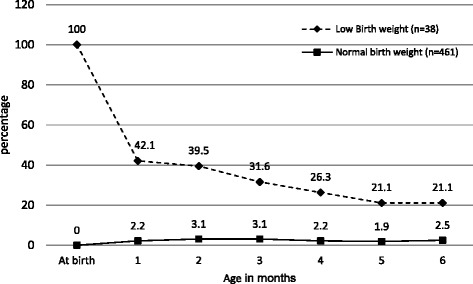
Prevalence of underweight (WAZ < -2SD) among normal birth weight and low birth weight infants

The underweight (WAZ < -2 Z-score) rates for the normal birth weight infants remained between 2.0 % and 3.1 % during the first 6 months of life, with 97 % above -2 Z-score. In contrast, the underweight rates for the LBW infants decreased from 42 % at 1 month to 22 % at 6 months of age, so 77 % were above -2 Z-score (Fig. 4).

## Discussion

This study was carried out to document the progress of a small-scale programme that has been promoting optimal breastfeeding practices through peer counsellors in urban and rural settings in Bangladesh for several years. High rates of early initiation of breastfeeding and of exclusive breastfeeding were seen in the programme area. At the same time, there were no signs of growth faltering in the infants of the counselled mothers during the first 6 months.

As peer counsellors were requested to give more emphasis on explaining the importance of initiating breastfeeding within the first hour of birth, the current reported initiation rate is higher at 94 % versus 79 % in 2011 [32] and substantially higher than the 57 % initiation rates reported in the Multiple Indicator Cluster Survey of 2013–14 [33]. Our latest rate of 94 % exclusive breastfeeding prevalence at six months was however consistent with our earlier findings of 88 % [32] and comparatively much higher than that reported in the 2014 national survey (55 % exclusive breastfeeding among 0-6 month infants) [13]. These results thus seem to suggest that the peer counselling intervention may be making a difference in the breastfeeding rates in these areas. This is a likely possibility as our results are also similar to those obtained from a cluster-randomized evaluation of a large non-government project in Bangladesh, where the exclusive breastfeeding rate in the intervention areas during 5 years rose to 86 % compared to 51 % in comparison areas [34]. It is of interest to note, however, that the intervention package in the latter project had huge resources and included a combination of group and inter-personal counselling by staff and outreach workers/paid volunteers, mass media communications, community mobilization and advocacy to create an enabling environment for optimal IYCF practices.

Also noteworthy is that our facility-based delivery rates at 60 % were higher than the 40 % national rates [13]. This may have happened because peer counsellors encouraged mothers to go to health facilities for delivery, explained the risks of home delivery by untrained birth attendants, and gave referral slips when required for health facilities where TAHN had already established linkages and staff were supportive of breastfeeding.

Understandably, some of the mothers did have difficulties in continuing exclusive breastfeeding, and their reasons are mentioned in Table 5. In other cases, though, it was the doctors or the grandparents who advised other milk or other liquids or foods to be given in addition to breastmilk. Some of the quoted reasons were similar to those that have been reported in one of our earlier reports [35] and other studies elsewhere [36, 37] which indicate that more efforts will be required to identify and target vulnerable mothers and to provide them (and their families) with additional information and support so that they feel more confident in their ability to breastfeed exclusively.

As our peer counsellors had expressed concerns regarding difficulties in breastfeeding by mothers of the LBW infants, we tried to find reports of breastfeeding/ breastfeeding counselling of LBW infants and found only three reports, all from the Philippines [20, 38, 39]. While the earlier study had reported that LBW infants were breastfed less compared to infants with normal birth weight [38], the later one showed that peer counselling of mothers of term LBW infants after hospital discharge could achieve 44 % exclusive breastfeeding at 6 months compared to 7 % in mothers who received only childcare counselling, and none in the controls [20]. The same authors also showed that those infants who were exclusively breastfed from birth to 6 months had very little diarrhoea compared to partially breastfed and non-breastfed infants and showed catch-up growth in the first 4 months [39]. So although the LBW group in our study was small, it is quite remarkable all the mothers managed to breastfeed their infants exclusively for 6 months.

When it comes to peer counselling, a comprehensive review of published research has shown that peer counsellors are effective in improving rates of breastfeeding initiation, duration and exclusivity while significantly decreasing the incidence of diarrhoea in infants and increasing lactation amenorrhoea [19]. The review’s authors further emphasize that “although interventions have focused primarily on models serving low-income women, this should not be interpreted as implying that only the low-income group benefits from peer counselling. Where healthcare resources are limited, the peer counsellors may be the only feasible means to provide breastfeeding education and support to prevent early childhood illness and growth faltering” [19]. And yet, peer counselling is an option, which unfortunately, has not been utilised widely in Bangladesh or in the South Asian region.

Looking at the growth studies, we have an earlier report from a rural area that showed Bangladeshi infants were shorter, lighter and thinner when compared with the WHO growth standards [40]. Our findings are consistent with these findings as growth and weight of both normal birth weight and LBW infants remained parallel to, but somewhat below the WHO child growth standards. At the same time, except for slower growth in the first month, no signs of growth faltering were seen in either group of infants. On the contrary, by 5 months of age the gap between the normal birth weight girls and those in the WHO standards had almost disappeared. On the whole, and especially since child undernutrition rates are so high in the country, these growth patterns are encouraging and suggest that peer counsellors’ supportive services may also be contributing towards prevention of growth faltering in both normal birth weight and LBW infants. Larger studies, however, will be required to confirm these findings. An additional contributory factor may be the lower incidence of illness in these infants (as per peer counsellor’s verbal reports), but that too, may be associated with better caring practices. It would be important to follow-up these children to monitor their complementary feeding patterns and examine its impact on their growth during early childhood.

We recognise several limitations of this assessment. We could not study a control group during the same period due to resource constraints. The number of LBW infants was much smaller than we had expected compared to the normal birth weight infants so we could not use any statistical tests for comparisons. We could not observe feeding practices at home and therefore relied on mother’s recall of infant feeding. However, as both 24-h recall on current visit and feeding practices during the last two visits were collected every month, we assumed this provided reliable information on infant feeding, and comparison between the two methods reduced recall bias which as others have pointed out, is minimal [41–43]. Due to scarce resources and logistic setbacks, it was not possible to measure infants’ lengths. We were thus unable to use length-for-age, length increments and stunting as indicators of nutritional status. Mothers’ weights and heights were not recorded during pregnancy so the impact of their nutritional status, particularly weight gain during pregnancy, which is an important determinant of birth weights, could not be assessed. Our analyses did not take into account any confounders (such as a mothers’ nutritional status) that may have influenced the outcomes, and that is probably another limitation of this study. Finally, due to absence of birth weights of those who had delivered away from the programme area (particularly in other villages), it is difficult to extrapolate the results, and further investigation in larger programme settings would be needed.

The main strength of this study, however, is that it comes from programme areas where we have been working for 15 years, and each cohort of mothers and babies is followed up for 18 months. There is regular interaction, and good rapport between peer counsellors, mothers and their families and programme staff, so mothers share their views freely. Furthermore, we have presented information on all LBW infants, whether delivered vaginally or surgically, preterm or term, of primiparous or multiparous mothers. The analysis thus was truly representative of an actual programme setting and could act as a model to show that small-scale programmes can also achieve good results, and therefore coverage area should not be a deterrent for undertaking them. Of course there could be other competing explanations for all the positive findings, such as the presence of the programme in these areas for so many years. Thus there may well have been a change in the community’s views towards exclusive breastfeeding, or more mothers may have grown up watching infants being exclusively breastfed, and being expected to do so themselves. It may also be argued that women who accept peer counselling could be different from those who do not, but we did not have many refusals in order to compare their characteristics. At the same time, it needs to be pointed out that the peer counsellors are basically extremely committed women. Their commitment has increased over time as they see the infants and young children of counselled mothers stay healthy and thrive, and their services are valued and respected by the community. It is not easy to find and keep such committed women, as often in rural communities women are not allowed to go from house to house, and be seen or talk to other men. The programme staff ensures that everything is explained clearly to the husbands and other significant family members when recruiting the peer counsellors, and also thank them for their support whenever they meet them.

Because of the dense population and low ratio of health staff to patients, it is well recognised that despite preventive health education being urgently needed, in actual practice time given for this is very limited [44]. This is more so where there are insufficient well child visits and domiciliary preventive health services. Frequent and targeted visits by community-based peer counsellors can be suggested, not only to encourage and support mothers to breastfeed exclusively, but also to promote and monitor infant growth, and to take timely action for improving their nutritional status.

## Conclusions

In the context of a well-structured programme setting, and under the described circumstances, it seems likely that appropriately trained and supervised community-based peer counsellors could assist in encouraging and helping mothers of both normal birth weight and LBW infants to initiate breastfeeding within 1 hour and to continue exclusive breastfeeding until 6 months of age. Our data suggest that they may also have contributed towards prevention of growth faltering of the infants.

## Abbreviations

WHO, World Health Organization; LBW, low birth weight; WAZ, weight-for-age Z score; SD, standard deviation; TAHN, training & assistance for health & nutrition foundation
